# Evolution Characterization and Pathogenicity of a Porcine Reproductive and Respiratory Syndrome Virus Isolate from a Pig Farm in Shandong Province, China

**DOI:** 10.3390/v14061194

**Published:** 2022-05-31

**Authors:** Yulin Xu, Xiaojing Ji, Chunyu Fu, Dong Hu, Heng Pang, Tingting Wang, Chuangang Li, Gang Wang, Jun Peng

**Affiliations:** 1 College of Veterinary Medicine, Shandong Agricultural University, Tai’an 271000, China; xuyulin2022@126.com (Y.X.); jixiaojing2022@126.com (X.J.); fuchunyu2022@126.com (C.F.); hudong202200@126.com (D.H.); pangheng2022@126.com (H.P.); wangtingting202200@126.com (T.W.); lichuangang2022@126.com (C.L.); 2 Shandong Provincial Key Laboratory of Animal Biotechnology and Disease Control and Prevention of China, East China Scientific Experimental Station of Animal Pathogen Biology of Ministry of Agriculture and Rural Affairs of China, Tai’an 271000, China

**Keywords:** porcine reproductive and respiratory syndrome virus, novel classical isolate, genetic stability, low pathogenicity

## Abstract

In recent years, porcine reproductive and respiratory syndrome virus (PRRSV) strains have been experiencing extensive recombination in Chinese swine farms. This recombination usually happens in NADC30/34 strains and highly pathogenic (HP) PRRSV strains. This study identified a new PRRSV isolate that shared 99% and 99.1% nucleotide identity with CH-1a and CH-1R at the genomic level, respectively. After purification by viral plaque assay, this isolate was named PRRSV CSR1801. The isolate did not experience any recombination with other PRRSV strains common in swine herd epidemics in China, which means it still maintains the stable features of the classical PRRSV strain and did not easily recombine with other PRRSV strains. Further analysis of the pathogenicity of the PRRSV isolate CSR1801 was performed in piglets. The results indicated that none of the inoculated piglets showed the typical clinical manifestations of PRRS, which presented with runny noses, rough back hair, rectal temperatures always below 40.5 °C, and no deaths. Additionally, no obvious histopathological lesions such as severe interstitial pneumonia could be observed in the lungs of the piglets. Hence, the PRRSV isolate CSR1801 should be classified as a classical-like PRRSV strain. This classical PRRSV strain showed genetic stability and maintained low pathogenicity. This study may provide new clues for further understanding the genetic evolution and pathogenicity of PRRSV and may also be an important reference for the prevention and control of PRRS in swine farms.

## 1. Introduction

Porcine reproductive and respiratory syndrome (PRRS) has become one of the most economically significant pig diseases all over the world since it was first reported in 1986 and 1990 in North America and Europe, respectively [1]. PRRS virus (PRRSV), horse arteritis virus, simian hemorrhagic fever virus, and lactate dehydrogenase-elevating virus are all members of the *Arteriviridae* family. The PRRSV genome comprises a positive-sense single-stranded RNA of approximately 15 kb in length and encodes 10 open reading frames (ORFs) [2]. The ORF1 (ORF1a and ORF1b), which encodes the RNA replicase polyprotein in the PRRSV genome, accounts for approximately 80% of the entire genome. The enzyme can be cleaved into 13 non-structural proteins with different biological functions (Nsp1α, Nsp1β, Nsp2–Nsp12), of which Nsp2 shows the greatest variation [3].

PRRSV was recognized as two distinct virus species (PRRSV-1 and PRRSV-2) instead of two genotypes (genotype I and genotype II) by the International Committee on Taxonomy of Viruses in 2016, although many researchers usually evaluate the effect of cross-protection of one PRRSV MLV against both PRRSV-1 and PRRSV-2 [4]. In China, almost all of the PRRSV isolates belong to PRRSV-2, and the first PRRSV isolate was reported in 1996 and was named CH-1a. Since highly pathogenic PRRSV (HP-PRRSV) appeared in 2006 [5,6,7], CH-1a has been considered to represent classical PRRSV strains (C-PRRSV) and is rarely isolated from clinical samples. The main difference between HP-PRRSV and C-PRRSV is the absence of the discontinuous deletion of 1+29 non-contiguous amino acids at positions 481 and 533–561 of Nsp2 in HP-PRRSV. In recent years, most PRRSV isolates from swine farms have appeared with new deletion sites on Nsp2, and the number of deletions is constantly changing, e.g., the NADC30 strain following the importing of breeding pigs came to China and underwent mutations or recombination, resulting in variant NADC30-like viruses characterized by the 131-aa discontinuous deletions in Nsp2 (a 111-aa deletion at position 322–432, a 1-aa deletion at position 483, and a 19-aa deletion at position 504–522 corresponding to the NADC30 complete sequence); as well as including the discontinuous deletion of 1+29 aa similar to HP-PRRSV, the other continuous deletion of 120 amino acids was found between aa 628 and 747 in the Nsp2 coding region of a PRRSV JX2014T2 isolate [8]. These variant PRRSVs cause highly variable clinical outcomes ranging from inapparent to severe.

Although numerous variant PRRSVs have been reported, most recombination happens among NADC30 and HP-PRRSV or mutations/deletions in HP-PRRSV strains. The impact of these strains varies among swine herds, but the consensus is that the pathogenicity trends to be intermediate between the parental strains [9,10,11]. PRRSV variation in C-PRRSVs, i.e., PRRSV Ch-1a-like or CH-1R-like, have been found to co-exist in Chinese swine herds [12,13], but the related reports are sparse and the pathogenicity of the C-PRRSV-like isolates has not been evaluated. Herein, we report a novel C-PRRSV variant isolated from a diseased pig farm in Shandong province, China, which shared 99% and 99.1% nucleotide identity with CH-1a and CH-1R at the genomic level, respectively, and caused mild PRRS symptoms during the infection in 28-day-old PRRSV-free piglets. These results may provide valuable clues for further understanding the genetic evolution of PRRSV and the prevention and control of PRRS.

## 2. Materials and Methods

### 2.1. Sample Collection and Virus Isolation

PRRSV-positive lung samples were collected from a PRRS suspected pig farm uninoculated with PRRSV vaccine from Shandong Province, China, in 2018. These samples were homogenized with Dulbecco’s modified Eagle medium (DMEM, Life Technologies Corp., Grand Island, NY, USA). The treated samples were inoculated after passing through 0.22 µm filters to MARC-145 cells. The inoculated cells were maintained in DMEM supplemented with 2% fetal bovine serum (FBS) and 100 U/mL of penicillin plus 100 μg/mL streptomycin at 37 °C in a 5% CO_2_ atmosphere. When approximately 80% of the cells exhibited a cytopathic effect (CPE) in the fourth generation, the supernatant of the virus culture was collected after two freeze-thaw repeats and stored at −80 °C for further use. 

### 2.2. Plaque Assay

For purification of the isolated virus, a plaque assay was performed using MARC-145 cells as previously described [14]. Briefly, the stock virus was 10-fold serially diluted and overlaid on a ~90% confluent monolayer of MARC-145 cells cultured in 6-well cell culture plates (0.5 mL/well). After a 1.5 h adsorption period at 37 °C, the plates were washed with sterile PBS and overlaid with melted agarose (2 mL/well). Two days of infection later, 2 mL/well of 0.01% neutral red staining solution (Solarbio, Beijing, China) was added for staining at 37 °C for 3 h. Then, a clear and uniform plaque was picked out and inoculated with new MARC-145 cells in 96-well cell culture plates. When approximately 80% of the cells displayed visible CPEs, the cells were frozen at −80 °C and thawed twice. After centrifugation at 10,000× *g* for 5 min, the supernatant was subjected to two additional rounds of plaque purification.

### 2.3. Immunofluorescence Assay (IFA)

MARC-145 cells were infected with PRRSV strain CSR1801 at a multiplicity of infection (MOI) of 0.1 when the cells reached approximately 90% confluence. At 24 h post-infection (hpi), the cells were fixed with 4% paraformaldehyde and then permeabilized with 0.3% Triton X 100. After three washes with PBS, they were blocked with 5% fetal bovine serum albumin at 37 °C for 1 h. The cells were incubated overnight at 4 °C with the monoclonal antibody (mAb) against PRRSV N protein at a dilution of 1:5000. Then, the cells were incubated with Alexa Fluor 488-conjugated goat anti-mouse IgG (Proteintech, Wuhan, China) at 37 °C for 1 h. After a final washing, the cells were observed using a fluorescent microscope (Leica, SPE, Buffalo Grove, IL, USA).

### 2.4. Viral Genome Extraction and RT-PCR Amplification

Total RNA was extracted from the CSR1801-infected MARC-145 cells’ supernatants using TRIzol reagent (Cwbio, Beijing, China). The full-length genome of the CSR1801 virus was obtained by amplifying each gene fragment using RT-PCR based on the specific primers (Table 1) according to a previous study [15]. Briefly, 2 μL total RNA was mixed with 1 μL 6-mer random primers (50 μM) (TaKaRa, Dalian, China), 1 μL deoxynucleoside triphosphate (dNTP) mixture (10 mM), and 6 μL RNase-free water (TaKaRa), incubated at 65 °C for 5 min, and then chilled on ice immediately. Then, 4 μL of 5× Primer Buffer, 0.5 μL RNase inhibitor (40 U/μL), 1μL PrimeScript RTase (200 U/μL), and 4.5 μL RNase-free water (TaKaRa) were added to the 10 μL reaction mixture. The reaction was incubated at 30 °C for 10 min and 42 °C for 1 h to reverse transcribe to cDNA then at 95 °C for 5 min to inactivate the reverse transcriptase. The 50 μL PCR reaction mix consisted of 25 μL PrimeSTAR Max Premix (2×), 2 μL of corresponding primers, 2 μL cDNA template, and 21 μL RNase-free water (TaKaRa, Dalian, China). PCR was performed in 30 cycles of 98 °C for 10 s, 60 °C for 15 s, and 72 °C for 1–2 min. The PCR product was purified using a gel extraction kit (TransGen, Beijing, China) and cloned into the pEASY-T1 cloning vector (TransGen). The cloned product was transformed into DH5α competent cells (TransGen) and the positive clones were picked for sequencing (Sangon, Shanghai, China). The obtained sequences were assembled using Lasergene software (DNASTAR Inc., Madison, WI, USA).

### 2.5. Phylogenetic and Recombination Analysis

The evolutionary relationship of the CSR1801 isolate with other represented Chinese PRRSV isolates was analyzed using MegAlign in the Lasergene software (DNASTAR). Phylogenetic trees were constructed using the MEGA software (version X) using the neighbor-joining method, and the reliability of the tree was assessed by bootstrap analysis with 1000 replications. The nucleotide and deduced amino acid (AA) sequences were aligned using the MegAlign program of the DNASTAR software (DNASTAR) to determine the sequence homology. A phylogenetic tree was constructed using the MEGA5 software with the neighbor-joining method; bootstrap values were calculated for 1000 replicates for alignment with multiple sequences of representative PRRSV sequences available in GenBank.

Furthermore, the software MegAlign was used to compare the full-length gene sequences of VR2332, CH-1R, JXA1, NADC30, and HLHDZD32-1901 (NADC34-like) strains. The above file was saved and homologous recombination analysis was performed using Simplot software (version 3.5.1). The CSR1801 strain was selected as the recombined parent sequence and Do BootScan was used to carry out a homologous recombination analysis.

### 2.6. Animal Experiments 

The experimental protocol for the pig studies was approved by the Shandong Agri-cultural University Animal Care and Use Committee (Approval Number: # SDAUA-2019-022). The animal experiments were conducted in strict accordance with the Chinese Regulations on Laboratory Animals and the Guidelines for the Care of Laboratory Animals (the Ministry of Science and Technology of the People’s Republic of China).

A virus challenge experiment in piglets was performed to evaluate the pathogenicity of the PRRSV CSR1801 strain. A total of 10 28-day-old PRRSV- and antibody-double-negative piglets, as assessed by RT-PCR and enzyme-linked immunosorbent assay (ELISA; IDEXX PRRS X3, Westbrook, ME, USA), were randomly divided into two groups. The piglets in group 1 (*n* = 5) were all nasally inoculated with 2 mL/pig of the PRRSV CSR1801 strain at a dose of 1 × 10^5^ TCID_50_. The piglets in group 2 (*n* = 5) were inoculated with the cell culture medium as the negative control. The piglets were monitored daily for clinical signs of PRRS, including fever (assessed by rectal temperature), respiratory symptoms (scored as described previously [16]), and abnormal behavior; the mental status, appetite status, and fecal status of the animals were also monitored to detect clinical signs of PRRS. Serum samples were collected from the piglets at 0, 3, 5, 7, 10, 14, and 21 days post-inoculation (dpi) and used in assays to measure the cytokine level and viral load. At 21 dpi, piglets were euthanized to collect lungs, lymph node, and spleen. The collected sample tissues were fixed in 4% formalin buffer and used for histopathological examination via hematoxylin and eosin (H&E) staining.

### 2.7. Real-Time Quantitative PCR (RT-qPCR)

The viral loads of PRRSV in the sera collected at serial timepoints (0, 3, 5, 7, 10, 14, and 21 dpi) were determined by conducting absolute RT-qPCR as reported previously [17]. First, a standard curve was established according to the results of six 10-fold gradients of recombinant plasmid amplified with the primers: forward, 5′-ACCAGGCGTTTCGCATCT-3′; and reverse, 5′-ACTCTCTGCACTCACGGAAGG-3′. The extracted total RNA was then subjected to reverse transcription and RT-PCR, and the virus RNA quantity of the sample was calculated from a linear extrapolation of the Ct value plotted against the standard curve.

### 2.8. Enzyme-Linked Immunosorbent Assay (ELISA) 

The concentrations of IFN-α, IFN-γ, IL-10, and TGF-β1 in pig sera collected at serial timepoints (0, 3, 5, 7, 10, 14, and 21 dpi) were detected using commercial kits for IFN-α, IFN-γ, IL-10, and TGF-β1 (Mlbio, Shanghai, China) detection in accordance with the manufacturers’ instructions. The concentrations of the cytokines were calculated according to the corresponding OD_450_ value based on the corresponding established standard curve.

### 2.9. Histopathological Examination

Histopathological examinations of the collected lungs were performed using the H&E method. Briefly, the dried tissue sections were passed through the following alcohol gradient: xylene I (15 min), xylene II (15 min), alcohol xylene (1:1) (5 min), 100% alcohol (3 min), 90% alcohol (3 min), 80% alcohol (3 min), 70% alcohol (3 min), and deionized water (4 min). The sections were then dyed with hematoxylin for 5 min and rinsed for 1 s. The sections were subsequently covered with 1% hydrochloric acid alcohol for 2–3 s and flushed for 16 min. Next, the slices were placed in 85% alcohol for 2 s, 1% eosin for 3 s, dipped in 95% alcohol, 95% alcohol for 3 min, 100% alcohol for 2 min, and xylene for 10–30 min. Finally, the slices were sealed with neutral resin and the pathological changes in the lungs were observed under a light microscope (Optika, Ponteranica, Italy).

### 2.10. Data Analysis

The data are expressed as the mean ± standard deviation (X ± SD), Duncan’s multi-sample test was used to analyze the differences between the groups, and SPSS 19.0 was used for statistical analysis. *p* < 0.05 was considered statistically significant.

## 3. Results

### 3.1. Virus Isolation and Identification

**Figure 1:**
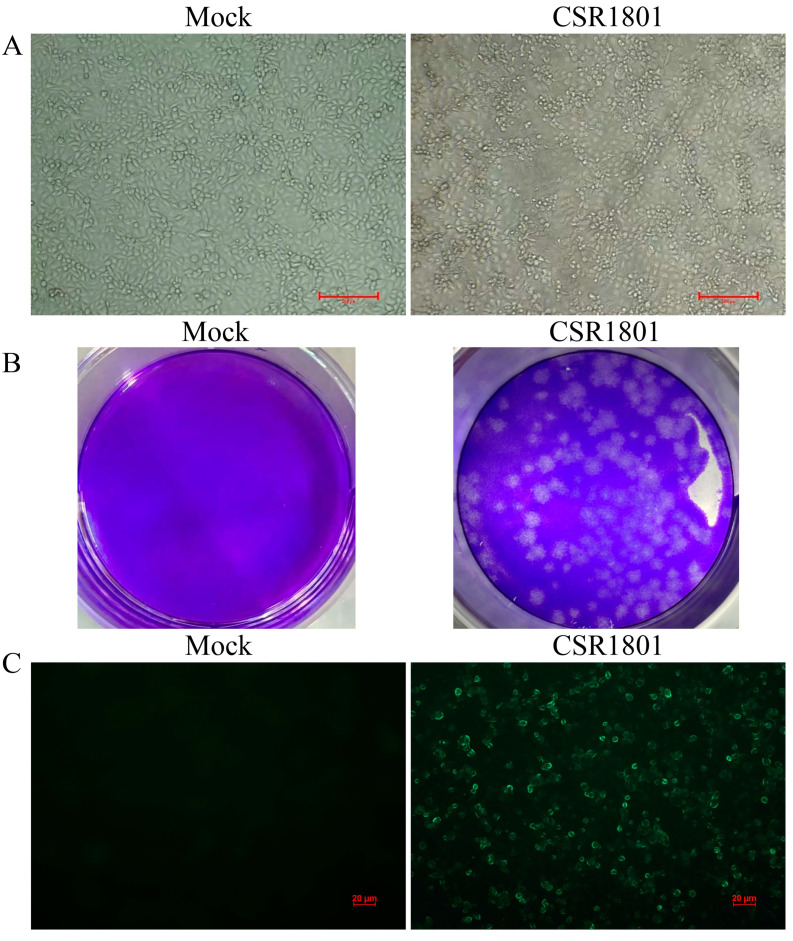
The isolation and identification of PRRSV strain CSR1801 by CPE, virus plaque, and IFA, respectively. (**A**) The typical CPE, including rounding, reduction and swelling in the cell, could be observed in the MARC-145 inoculated with the fourth generation of virus isolate. (**B**) The plaques were shown in plaque assay at the 500-fold dilution of the virus stock. (**C**) The PRRSV strain CSR1801 separated from the virus plaques was amplified and identified by immunofluorescence assay using the monoclonal antibody against PRRSV N protein.

MARC-145 cells were inoculated with the grinding fluid of PRRSV-positive lung samples, and typical PRRSV-induced CPE appeared at 48 h after inoculation from the fourth-generation virus (Figure 1A). One of the viral plaques was picked out from the virus stock by a virus plaque assay as shown in Figure 1B and was subsequently amplified in MARC-145 cells and named PRRSV strain CSR1801. Then, the presence of PRRSV was confirmed by IFA staining with a PRRSV-specific mAb against the virus N protein. As shown in Figure 1C, after 24 h inoculation of MARC-145 cells with the CSR1801 strain, PRRSV-specific fluorescence was observed in the field of vision; by contrast, MARC-145 cells in mock-infection could not react with the mAb against N protein.

### 3.2. Analysis of Full-Length Genomic Sequence

**Figure 2:**
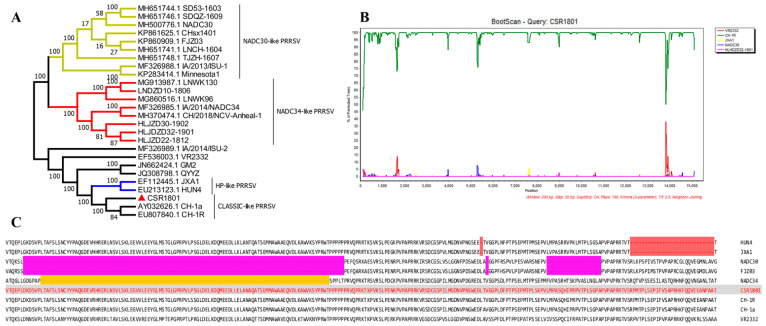
Phylogenetic and recombinant analysis of the PRRSV CSR1801 strain. (**A**) The phylogenetic tree of the whole genome indicated the CSR1801 strain was classified into the CLASSIC-like PRRSV. (**B**) The recombinant analysis between the CSR1801 strain and VR2332, CH-1R, JXA1, NADC30, and HLHDZD32-1901 (NADC34-like) strains using RDP4 and SimPlot software showed that the CSR1801 strain did not recombine with the other types of PRRSV. (**C**) The alignment of the deduced Nsp2 amino acid sequence between the PRRSV CSR1801 strain and other types of PRRSV strains showed that the CSR1801 strain did not delete any amino acids and was consistent with the CLASSIC-like PRRSV, while it was significantly different from HP-like, NADC30-like, and NADC34-like PRRSVs.

The full-length genome of the PRRSV CSR1801 strain was determined by segmental gene sequencing and splicing, which is 15,219 bp excluding the poly (A) tail (GenBank accession number: OM743305). To investigate the genetic relationships between the PRRSV CSR1801 strain and other PRRSV strains, the whole genome sequence of the CSR1801 strain and the representative PRRSV reference strains or vaccine strain registered in GenBank were used for phylogenetic tree analysis. Based on the phylogenetic tree result of full-length genome sequence analysis, the PRRSV isolate CSR1801 was classified into the CLASSIC-like PRRSV group (Figure 2A), which shared 99% and 99.1% nucleotide identity with CH-1a and CH-1R at the genomic level, respectively. Meanwhile, RDP4 and SimPlot software were used to analyze the recombination within the whole genome sequences between the CSR1801 strain and other representative PRRSV strains. The results showed that there was no gene fragment recombination among the strains of CSR1801, VR2332, CH-1R, JXA1, NADC30, and HLHDZD32-1901 (NADC34-like), as shown in Figure 2B. Moreover, to further characterize the CSR1801 strain, the Nsp2 amino acid sequences were aligned with the PRRSV strains, including HP-PRRSV, classical PRRSV, NADC30, and NADC34. Nsp2 possesses highly variable features and is recognized as a molecular marker in HP-PRRSV-like and NADC30/34-like strains, which are currently epidemic in swine farms. Sequence alignment suggested that Nsp2 amino acids of the CSR1801 strain did not contain recombination or deletion positions (Figure 2C). The analysis of the full-length genome sequence and Nsp2 amino acid sequences suggested that classical PRRSV strains are still epidemic but have weak recombination characteristics in some swine farms.

Finally, we analyzed the amino acid mutation of the CSR1801 strain compared with CH-1R and CH-1a strains, which showed that the ORF1a (2503 aa) amino acid mutation of the CSR1801 strain has 23 different loci—155, 235, 402, 509, 510, 582, 851, 860, 867, 997, 1023, 1259, 1307, 1445, 1508, 1549, 1640, 1643, 1755, 2166, 2273, 2341, and 2410; 12 mutations are identical to CH-1a but not to CH-1R—227, 560, 764, 964, 969, 1245, 1630, 2067, 2109, 2295, 2441, and 2448; 18 mutations are identical to CH-1R but not to CH-1a—573, 780, 865, 1015, 1038, 1193, 1254, 1429, 1522, 1523, 1543, 1705, 1742, 1797, 1994, 2063, 2105, and 2241. A detailed analysis of the corresponding amino acid changes is shown in Table 2. The ORF1b (1457 aa) amino acid mutation of the CSR1801 strain is different from that of the CH-1R and CH-1a strains at 9 loci—2619, 2838, 2933, 3426, 3433, 3537, 3783, 3926, and 3936; ORF2 (257 aa) has 3 loci—4021, 4178, and 4182; ORF3 (255 aa) has 3 loci—4219, 4384, and 4402; ORF4 (179 aa) has 1 locus—4522; ORF5 (201 aa) has 3 loci—4546, 4606, and 4683; ORF6 (175 aa) has 4 loci—4791, 4806, 4816, and 4881; and ORF7 (124 aa) has 3 loci—4944, 4975, and 5017. These results demonstrate that the current classical PRRSV strains epidemic in swine farms have experienced mutations and do not show any recombination features with HP-PRRSV or NADC30/34 strains.

### 3.3. Viral Load in Sera

**Figure 3:**
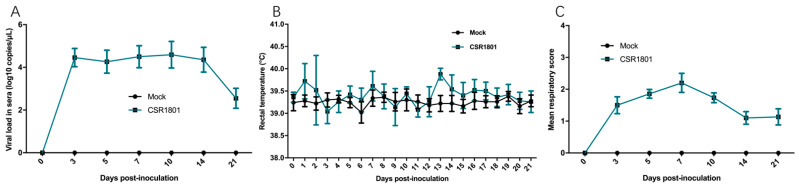
Virus load in the peripheral blood, rectal temperature, and respiratory score. The piglets in group 1 (*n* = 5) were all nasally inoculated with 2 mL/pig of the PRRSV CSR1801 strain at a dose of 1 × 10^5^ TCID_50_. The piglets in group 2 (*n* = 5) were inoculated with the cell culture medium as the mock-inoculated control. (**A**) The virus amounts in sera collected at serial timepoints (0, 3, 5, 7, 10, 14, and 21 dpi) from the CSR1801-inoculated and mock-inoculated piglets. The data represent the mean values (±SD) of the log10 virus copies/mL determined by conducting absolute RT-qPCR. (**B**) Rectal temperature changes in piglets after virus infection. The daily average temperatures of the animals in the group infected with the CSR1801 strain and the mock group piglets are shown. (**C**) Mean respiratory score from the CSR1801-inoculated and the mock-inoculated piglets at serial timepoints (0, 3, 5, 7, 10, 14, and 21 dpi). Each point represents the mean values (±SD) generated from all the pigs at 0–21 dpi.

The viral titers of PRRSV in the sera collected from piglets at regular intervals were detected by RT-qPCR. The results showed that the PRRSV CSR1801 isolate infection induced a low level of viremia. The viral titer peaked at 3 dpi, remained constant at about 4.2 log copies per microliter until 14 dpi, and declined rapidly to near zero after 14 dpi (Figure 3A), indicating the end of viremia.

### 3.4. Clinical Symptoms and Histopathological Lesion

In general, the piglets inoculated with the PRRSV CSR1801 isolate showed mild clinical signs. In the animal challenge experiment, the rectal temperature of piglets inoculated with PRRSV CSR1801 strain increased to 39.7 °C at two dpi and it always fluctuated around 39.5 °C the rest of the time. The rectal temperature of the mock-inoculated pigs was relatively stable and almost always about 39.0–39.2 °C (Figure 3B). Mild clinical signs were observed in the challenged pigs. Their respiratory disease scores are summarized in Figure 3C. All five piglets showed mild dyspnea when stressed or at rest from three dpi to 10 dpi. Other symptoms included a runny nose and rough back hair in infected piglets from eight dpi to 16 dpi, but all were in good mental status and showed a good appetite. There were no deaths. By contrast, the mock-inoculated piglets behaved normally without any clinical signs throughout the experiment.

**Figure 4:**
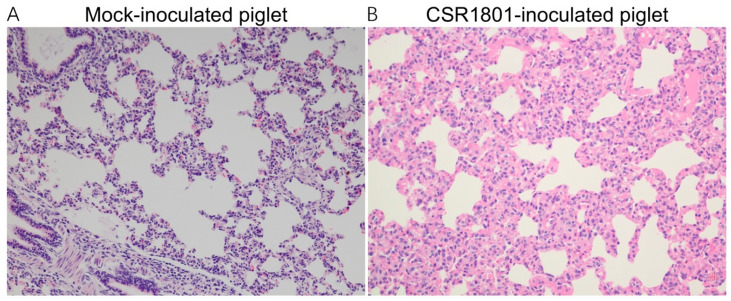
Histopathological results. The piglets in group 1 (*n* = 5) were all nasally inoculated with 2 mL/pig of the PRRSV CSR1801 strain at a dose of 1 × 10^5^ TCID_50_. The piglets in group 2 (*n* = 5) were inoculated with the cell culture medium as the mock-inoculated control. Histopathological examination of lung tissue from the PRRSV CSR1801—and of the mock-inoculated piglets at 21 dpi was performed using routine hematoxylin and eosin staining method, respectively. The results showed a mild lymphocytic interstitial pneumonia in lungs from the PRRSV CSR1801-inoculated piglets. Magnification, 200×.

Lymphocytic interstitial pneumonia characterized by a diffuse interstitial proliferation could be observed in the lungs in the PRRSV CSR1801 strain-inoculated piglets (Figure 4), which was consistent with the results of a previous study [18], while the mock-inoculated piglets showed normal lung structure.

### 3.5. Concentrations of the Corresponding Cytokines in Sera

**Figure 5:**
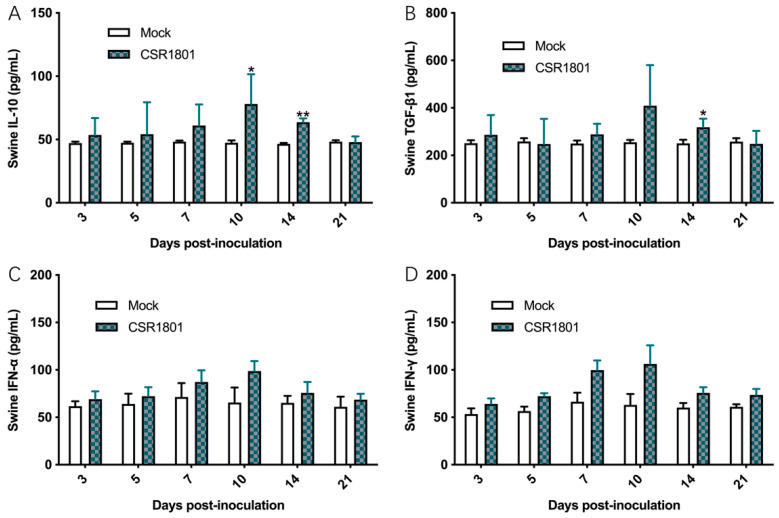
Levels of cytokines in peripheral blood of piglets collected at serial timepoints (0, 3, 5, 7, 10, 14, and 21 dpi). The piglets in group 1 (*n* = 5) were all nasally inoculated with 2 mL/pig of the PRRSV CSR1801 strain at a dose of 1 × 10^5^ TCID_50_. The piglets in group 2 (*n* = 5) were inoculated with the cell culture medium as the mock-inoculated control. The concentrations of swine IL-10 (**A**), TGF-β1 (**B**), IFN-α (**C**), and IFN-γ (**D**) were determined by the commercial ELISA kit. Each point represents the mean value (±SD) generated from all pigs in one group at the indicated timepoints post-inoculation. * *p* < 0.05; ** *p* < 0.01.

In the animal experiment, the concentrations of the corresponding swine cytokines, including IFN-α, IFN-γ, IL-10, and TGF-β1 in pig sera were determined by ELISA. Ten days after the challenge, the concentration of IL-10 did not change significantly. At 10 dpi and 14 dpi, the concentration of IL-10 in the CSR1801-inoculated piglets was significantly higher than that of the mock-inoculated piglets and then decreased rapidly (Figure 5A). The characteristic of TGF-β1 change was similar to that of IL-10, and its concentration in the virus-inoculated piglets was significantly higher than that of the control group at 14 dpi (Figure 5B). On the other hand, as cytokines play a role in cellular immunity, the levels of IFN-α and IFN-γ from the CSR1801-inoculated piglets was elevated at seven dpi and peaked at 10 dpi, then decreased to normal levels thereafter (Figure 5C,D), which demonstrates the similar change characteristics. 

## 4. Discussion

The PRRSV epidemic strains in China have experienced recombination since 2015 due to the introduction of the PRRSV NADC30 strain from North America that has undergone genetic exchange with the HP-PRRSVs in China [19,20,21], producing a series of PRRSV NADC30-like strains that have affected most of the swine farms in China for ~5 years [10,11,22,23]. In 2017, another PRRSV strain, which originated from NADC34 from North America, called NADC34-like, was first detected in a swine farm in Liaoning Province and then in Fujian Province [24,25], indicating that NADC34-like PRRSV might have the potential ability to develop an endemic strain in swine farms in China. The ratio of NADC34-like positives increased to 11.5% and 28.6% in 433 PRRSV-positive clinical samples from eight provinces in 2020 and 2021 [26]. A common characteristic of both NADC30 and NADC34 is strong recombination ability, and the recombination happens among NADC34-like, NADC30-like, or HP-PRRSV strains from local swine farms in China [26]. No recombination between NADC30/NADC34 and classical PRRSV has been reported in China. Maybe the weak recombination ability of classical PRRSV strains leads to its low positive percentage in the positive swine farms.

In our study, the PRRSV CSR1801 strain still shows the classical PRRSV features, and no recombination or deletion was found in the amino acids (AAs) of Nsp2, the most variable genes in the whole genome of PRRSV and NADC30/NADC34/HP-PRRSV [11,26,27,28]. However, as an RNA virus, the CSR1801 strain also experienced an evolution process. These mutations happened discontinuously and occasionally from ORF1 to ORF7, but whether these mutations are related to pathogenicity warrants further investigation.

The pathogenicity of the PRRSV CSR1801 strain for the piglets was mild and mainly manifested in clinical signs, rectal temperature, viral load, and lung lesions, which is consistent with the previous report [18]. Whether due to its genetic evolution or its pathogenicity, the PRRSV isolate CSR1801 showed the characteristics of a classical strain and should be classified as a genetically stable classical strain of PRRSV.

In China, PRRSV MLVs are used extensively in swine farms, and the MLV strains include VR-2332, R98, CH-1R, PC, JXA1-P80, HuN4-F112, GDr180, and TJM-F92, which can offer effective protection in homologous PRRSV strain challenges [29,30,31,32,33]. In recent years, the inactivated CH-1a vaccine has been widely used after one year of African swine fever introduction, although its efficiency is controversial. No study has reported that anyone PRRSV vaccine could completely protect against the main epidemic PRRSV strains of NADC30-like or NADC34-like in the field. According to the efficiency reports of commercial MLVs used in swine farms against different NADC30-like strains, these PRRSV MLVs showed limited or no cross-protection efficacy against some NADC30-like strains [21,23,34,35,36]. In particular, PRRSVs pools in swine farms increase viral recombination frequency and make the clinical prevalence and control of PRRS more complicated. Since the NADC30 invasion in 2015, variant strains that recombine with PRRSV MLVs originating from HP-PRRSV are often found in PRRSV-positive swine farms [11,37,38]. In these studies, although PRRSV NADC30 or NADC34 strains easily recombine and produce many NADC30-like or NADC34-like strains with local HP-PRRSV (-like) strains, the recombination of both NADC30 or NADC34 and the local classical PRRSV strain was not found. 

In summary, this study looked at a new classical PRRSV strain from a swine farm in Shandong province, China, which maintained stable features and did not recombine with other PRRSV strains (NADC30-like, NADC34-like, or HP-like strains) as is common in swine farms. This study may provide clues for further understanding the genetic evolution and pathogenicity of PRRSV and may also have important reference value for the efficient prevention and control of PRRS in swine farms.

## Figures and Tables

**Table 1:** Primers used for amplification in the full genome of the PRRSV isolate.

Fragments	Sequences of PCR Primers (5′–3′)	Position ^1^	Length of Amplicon
PRRSV-1F	ATGACGTATAGGTGTTGGCTCTATG	1–1894	1894 bp
PRRSV-1R	AGCGGCTGGGATAGCACTGCTAGGC		
PRRSV-2F	GGTGAGCATTGGACTGTCACTG	1700–3617	1918 bp
PRRSV-2R	TCTCGAGGATGCGTGGAACATC		
PRRSV-3F	GTCTGTTTACCAGGCGATTTGC	3427–5297	1871 bp
PRRSV-3R	CACAAAGCAACCAGGTAAGAGG		
PRRSV-4F	TTTCCCAACACGGCCTTACCCT	5103–7007	1905 bp
PRRSV-4R	TATCAGCAAAAGCTTCAAGTTTGG		
PRRSV-5F	ATCATGAGTCTCTGACTGGTGCCC	6777–8697	1921 bp
PRRSV-5R	CTTCTTCCCGCAATACTGTTTCTT		
PRRSV-6F	GAATTCTATGGCTGGAATAAATGG	8547–10407	1861 bp
PRRSV-6R	AACATAGCAATGAGAATCAAAACC		
PRRSV-7F	CACCGGTCCGTGGGTTCGCATCCT	10227–12097	1871bp
PRRSV-7R	AAAGGCTTTGCATGGACCCCATTT		
PRRSV-8F	GGAGTTCTCGTTGGATGACCCAGT	11847–13837	1991 bp
PRRSV-8R	ACAAAGAAAGCAATTGCGAGCAAC		
PRRSV-9F	CATCGTGGCTGTGTGTGTCAATTT	13607–15409	1803 bp
PRRSV-9R	AATTTCGGCCGCATGGTTCTCGCC		

^1^ The position is determined based on the representative strain ATCC VR-2332 (U87392).

**Table 2:** Detailed comparison of the amino acid changes in the CSR1801 strain with the CH-1a and CH-1R strains.

ORFs		aa Position	155	227	235	402	509	510	560	573	582
	PRRSV-Prototype										
ORF1a (2503 aa)	CH-1a		S	H	S	S	R	L	L	A	P
	CH-1R		S	Y	S	S	R	L	F	V	P
	CSR1801		G	H	G	L	C	H	L	V	S
			764	780	851	860	865	867	964	969	997
	CH-1a		A	K	D	R	C	R	V	P	E
	CH-1R		V	R	D	R	D	R	I	L	E
	CSR1801		A	R	N	Q	D	W	V	P	D
			1015	1023	1038	1193	1245	1254	1259	1307	1429
	CH-1a		N	S	E	R	T	L	E	W	R
	CH-1R		D	S	K	W	I	F	E	W	Q
	CSR1801		D	A	K	W	T	F	G	.	Q
			1445	1508	1522	1523	1543	1549	1630	1640	1643
	CH-1a		W	P	L	L	H	W	W	L	W
	CH-1R		W	P	F	F	Y	W	R	L	W
	CSR1801		R	S	F	F	Y	R	W	F	R
			1705	1742	1755	1797	1994	2063	2067	2105	2109
	CH-1a		A	S	W	H	S	W	A	S	S
	CH-1R		V	G	W	Y	R	R	S	F	F
	CSR1801		V	G	R	Y	R	R	A	F	S
			2166	2241	2273	2295	2341	2410	2441	2511	
	CH-1a		P	.	R	Q	P	W	W	R	
	CH-1R		P	R	R	.	P	W	R	C	
	CSR1801		S	R	.	Q	L	.	W	R	
ORF1b (1457 aa)			2619	2745	2837	2838	2933	2959	3023	3034	3130
	CH-1a		I	P	K	K	C	V	H	L	F
	CH-1R		I	L	E	K	C	A	Y	P	S
	CSR1801		V	L	K	E	S	V	Y	L	F
			3197	3247	3366	3424	3426	3433	3537	3551	3606
	CH-1a		E	V	L	R	I	D	V	K	S
	CH-1R		N	A	F	Q	I	D	V	E	T
	CSR1801		K	V	F	Q	V	G	A	K	T
			3775	3783	3847	3918	3926	3936			
	CH-1a		Y	W	V	K	A	Y			
	CH-1R		H	W	I	E	A	Y			
	CSR1801		H	.	V	E	T	N			
			3972	4021	4028	4052	4085	4102	4178	4182	
ORF2 (257 aa)	CH-1a		N	F	H	R	A	.	H	R	
	CH-1R		D	F	Y	L	T	W	H	R	
	CSR1801		N	Y	Y	L	T	W	Y	C	
ORF3 (255 aa)			4219	4252	4254	4303	4312	4334	4368	4384	4399
	CH-1a		N	G	D	Q	F	F	S	T	R
	CH-1R		N	S	E	R	V	Y	F	T	T
	CSR1801		D	S	E	Q	S	Y	F	I	R
			4402								
	CH-1a		A								
	CH-1R		A								
	CSR1801		T								
ORF4 (179 aa)			4522	4524							
	CH-1a		F	P							
	CH-1R		F	Q							
	CSR1801		L	P							
			4538	4546	4571	4606	4679	4683			
ORF5 (201 aa)	CH-1a		C	R	H	H	L	G			
	CH-1R		Y	R	Q	H	Q	G			
	CSR1801		Y	Q	Q	N	L	D			
			4791	4806	4816	4881					
ORF6 (175 aa)	CH-1a		S	Q	R	K					
	CH-1R		S	Q	R	K					
	CSR1801		L		Q	R					
			4909	4944	4946	4975	5017				
ORF7 (124 aa)	CH-1a		R	K	K	C	V				
	CH-1R		Q	K	N	C	V				
	CSR1801		Q	I	K	R	A				

## Data Availability

Not applicable.
